# Reply to the Editor Regarding Metal Hypersensitivity in Joint Arthroplasty: A Review Article

**DOI:** 10.5435/JAAOSGlobal-D-22-00017

**Published:** 2022-03-04

**Authors:** Johannes Michiel van der Merwe

**Affiliations:** Saskatoon, Canada

Hypersensitivity in total joint arthroplasty is a very complex and yet to be fully understood topic. In the article “Metal hypersensitivity in joint arthroplasty,”^1^ which was published in *JAAOS Global* in 2021, the author tried to review the current literature available to get a better understanding of the management and clinical diagnosis of metal hypersensitivity. An algorithm was proposed to deal with these complex cases. Although the clinical, radiographic, preimplantation, and postimplantation workup was the mainstay of the article, the author thought to include the pathogenesis to fully review the topic. Seeing that the pathogenesis of metal hypersensitivity is a difficult concept to grasp, the author tried to simplify the notion with figures. Unfortunately, Figure 2 led to confusion due to the combination of histopathological patterns and hypersensitivity reactions. The author would like to use this commentary as a medium to rectify the confusion caused by Figure 2.

Hypersensitivity reactions occur when a patient's immune system *responds* abnormally, which could potentially harm the patient. The four types of hypersensitivity reactions as per the Gell-Coombs classification can be classified as follows: type 1—immediate allergic reactions (an Immunoglobulin E [IgE] mediated reaction, which leads to cell degranulation); type 2—referred to as cytotoxic (antibodies that are specific to particular tissues within the body and cause destruction of cells in these tissues) Immunoglobulin M (IgM) and Immunoglobulin G (IgG), which mistakenly bind to surface antigens of cells in the body and lead to cellular destruction, inflammation, and impaired cellular function; type 3—immunocomplex-mediated reactions (an antigen binds to IgG to form an immune complex, which is subsequently deposited in tissues and leads to cell death and inflammation); and type 4—delayed or cell-mediated reactions and involve T-lymphocytes rather than antibodies. Metal hypersensitivity is a type 4 hypersensitivity reaction as per the Gell-Coombs classification.^2,3^

In Figure 2, reference was incorrectly made to Kren et al.^4^ who elaborated on four major histological patterns of the neosynovium/periprosthetic membrane. This can certainly lead to confusion, and therefore, it might be better suited to having an alternate description highlighting the four histopathological patterns and the relationship they have with hypersensitivity reactions. If the synovium is resected at the time of a joint arthroplasty, a new layer is formed (neosynovium); if this layer is between the implant and the bone, then it is named the periprosthetic membrane.

The four histopathological patterns include type 1—abrasion-induced type, type 2—infectious type, type 3—combined type, and type 4—fibrous type.

Macrophages and multinucleated giant cells with prosthesis wear features (wear particles) are the hallmark of type 1. It has been postulated that a type 4 hypersensitivity reaction is only probable in the type 1 histological pattern of the periprosthetic membrane where there are pronounced lymphocyte infiltrations. Most attention has been paid to the lymphocyte infiltration rather than macrophages or wear particles. The patterns of lymphocyte infiltrates do not yet have any specific prognostic or clinical value, and there is no clear relationship with type 4 hypersensitivity reactions. Currently, there are no histopathological findings in the neosynovium/periprosthetic membrane for type 4 hypersensitivity reactions. Excessive lymphocyte or plasmacellular infiltrates in a type 1 neosynovium is indicative of adverse tissue reactions in the presence of wear particles, and hypersensitivity should be considered if eosinophilic infiltrates or granulomas are present.

The hallmark of identifying type 2 is the presence of neutrophil granulocytes. Type 3 demonstrates features of bacterial infection (neutrophil granulocytes) and type 1, whereas type 4 does not exhibit features of type 1 or 2. In type 4 neutrophil granulocytes, lymphoplasmacytic infiltrates and abrasion wear particles are either absent or might occur sporadically.

In conclusion, the author would like to remove Figure 2 from the article to avoid additional confusion and hopefully the commentary would explain the reasoning behind it.

## References

[1] Van der Merwe JM. Metal hypersensitivity in joint arthroplasty. JAAOS Glob Res Rev 2021;5:1-8. 10.5435/JAAOSGlobal-D-20-00200 PMC7963506 33720103

[2] https://www.amboss.com/us/knowledge/Hypersensitivity_reactions/.

[3] Gell PGH Coombs RRA. The classification of allergic reactions underlying disease, in Coombs RRA Gells PGH, eds: Clinical Aspects of Immunology. Oxford, United Kingdom, Blackwell, 1963.

[4] Revised histopathological consensus classification of joint implant related pathology Author links open overlay panel V. Krenna1L.Morawietzb1G.PerinocH.KienapfeldR.AscherleG.J.HassenpflugfM.ThomsengP.ThomashM.HuberiD.KendoffjD.BaumhoerkM.G.KrukemeyerlS.NatumF.BoettnernJ.ZustinoB.KölbelaW.RütherpJ.P.Kretzerq…T.Gehrkej.

